# “We Live in Different Chicagos”: Racial/Ethnic Differences in the Neighborhood Affiliations of Young Men Who Have Sex with Men as Drivers of HIV Risk

**DOI:** 10.1007/s10461-025-04734-7

**Published:** 2025-04-24

**Authors:** Elizabeth A. McConnell, Michelle Birkett

**Affiliations:** 1 Department of Psychology, Palo Alto University, Palo Alto, CA USA; 2 Department of Medical Social Sciences, Feinberg School of Medicine, Northwestern University, Chicago, IL USA

**Keywords:** Racial disparities, HIV, Young men who have sex with men (YMSM), Social networks, Neighborhoods, Stigma

## Abstract

Racial disparities in HIV are well-documented and pervasive, particularly impacting Black young men who have sex with men (YMSM), and are not explained by differences in individual risk behaviors. The Network-Individual-Resource (NIR) Model of HIV Transmission and Prevention (Johnson et al. in AIDS Behav 14:S204–S221, 2010) suggests that focusing on the broader network-level environments of YMSM holds strong promise for identifying social-contextual factors that may drive these racial disparities. Empirically, numerous studies have demonstrated links between neighborhood-level factors and HIV risk among YMSM (Bauermeister et al. in J Sex Res 54:446–464, 2017). This mixed-methods study aimed to characterize racial differences in the broader contexts (i.e., neighborhood affiliations) within which YMSM meet their sex partners, which may in turn shape HIV risk. Using an innovative explanatory sequential design, multilevel network and geospatial data from an existing longitudinal cohort of YMSM in Chicago were visualized (Phase 1) and subsequently used to guide interviews with a subsample of participants in the broader parent study (Phase 2). Grounded theory was used to analyze interview data, leading to the identification of several salient differences in Black, White, and Latino YMSM’s neighborhood-level affiliations, including racial segregation and resource inequality, White spatial insularity, profound Black-White segregation, and Black and Latino neighborhood bridging. From a network perspective, these racial differences in neighborhood-level affiliations have strong potential to help explain the disproportionate burden of HIV among Black YMSM. Findings highlight the importance of attending to the social context within which individual risk behaviors take place and underscore the importance of broader structural change to address racial disparities in HIV.

## Introduction

Black men who have sex with men (MSM) have alarming rates of Human Immudodeficiency Virus (HIV) and are disproportionately impacted by the HIV epidemic [1–5]. The Centers for Disease Control and Prevention (CDC) estimates that between 2018 and 2022, Black MSM accounted for 31% of HIV cases among MSM, despite Black Americans making up only 12% of the US population [2]. Although research to date has identified multiple individual risk factors linked to HIV, Black MSM reported comparable or fewer of these relative to MSM of other racial groups: they were less likely to have unprotected anal intercourse, reported fewer sex partners, and were more likely to engage with HIV prevention efforts like testing and use of pre-exposure prophylaxis (PrEP). However, despite lower risk behaviors and higher engagement in HIV prevention services, Black MSM continue to demonstrate higher HIV prevalence and lower levels of awareness of their HIV + status [3–6]. Thus, research is shifting to focus on social contextual factors (e.g., poverty, stigma, discrimination) that may work together with individual behaviors to drive racial disparities in HIV [6, 7]. For example, if Black MSM meet sex partners in spaces with higher overall HIV prevalence, they may be more likely to become HIV + despite less risky individual behavior. In line with this multilevel approach, studies have highlighted a number of network- [6] and neighborhood-level [1] factors that may shape racial disparities in HIV among MSM.

## The Network-Individual-Resource Model of HIV Transmission and Prevention

The Network-Individual-Resource (NIR) Model of HIV Transmission and Prevention contends that HIV risk (and the means to decrease it) depends on the interplay of resources between individuals and their networks [8]. The NIR model identifies mental and tangible resources at the individual level as well as at multiple network levels: intimate dyadic, family, peers/community, and society. Mental resources include goal intentions to act safely, positive attitudes towards these actions, and perceived control over these actions. Tangible resources include material and energy stores such as income, possessions, and health [8]. Importantly, although an individual’s mental resources may support safe behavior related to HIV, this behavior is unlikely to occur unless the individual also has access to adequate tangible resources. Resources are linked to specific HIV prevention efforts at each level within the NIR model [8]. By focusing on resources at multiple levels and highlighting the interplay between individuals and networks, the NIR model shifts the focus for understanding HIV racial disparities from a solely individual level to an analysis of individuals in multiple social and societal/structural contexts. Although the NIR model provides a strong theoretical frame for multilevel approaches to addressing racial disparities in HIV, there is a relative lack of research on potential social-contextual drivers of racial disparities in HIV among YMSM. Guided by the NIR model, the current study addressed this gap by using a mixed-methods approach to characterize the neighborhood-level networks of Black, Latino, and White YMSM living in Chicago in order to identify potential racial differences in neighborhood affiliation, which in turn may structure racial/ethnic differences in HIV risk in these populations.

## Network Characteristics and Racial Disparities in HIV

Empirical research supports the importance of individual-level, venue-level, and neighborhood-level networks in understanding differential risk for HIV among YMSM. At the individual level, research documented that Black MSM had more dense, racially homophilous, and multiplex (i.e., sharing more than one type of relationship with a single person) sexual networks. In other words, the people they had sex with were more likely to be connected to other people they had sex with, they were more likely to be sex partners with other Black MSM, and they were more likely to have other kinds of relationships (e.g., friendship, acquaintance, drug use) with their sex partners [4, 9–16]. These differences in individual networks may directly impact HIV risk for Black MSM, as the combination of high-density sexual connections, racial homophily, and high prevalence rates of HIV among Black MSM mean that HIV will be transmitted more rapidly throughout the network [6, 17]. Further, multiplex relationships have been linked with higher HIV risk [9], and Black MSM may be more likely to encounter a UAI partner with HIV transmission potential [18]. However, research also suggests the relationships between individual network structure and HIV risk are complex. A recent systematic review found that the structure of social networks may be more predictive of HIV risk than sexual networks and the mechanisms linking social network characteristics to racial disparities in HIV are likely also complex. These authors suggest moving beyond a focus on individual sexual networks to examine how MSM are connected across higher-level (e.g., venue and neighborhood) networks, such as dynamics around bridging and racial segregation [6].

At the venue level, Black MSM showed high clustering around few venues [19–23] and HIV-positive Black MSM were linked by a small number of venues [21]. This may be due to feeling unwelcome at certain venues due to racial and/or sexual orientation discrimination, which can in turn lead to venue avoidance [24]. MSM of all races/ethnicities (including Black MSM themselves) reported that Black MSM were more difficult to meet than MSM of other races/ethnicities and that White MSM were more likely to feel welcome than MSM of color at MSM venues in San Francisco [14]. High clustering around venues can directly impact HIV risk, as it increases the likelihood that HIV will be transmitted more rapidly throughout a cluster once introduced [17]. It is also important to acknowledge that venues are geographically situated spaces, and venue-level patterns are likely related to the neighborhoods within which these venues are located.

At the neighborhood level, research on neighborhood characteristics highlights structural drivers of HIV risk. A recent systematic review examined the relationships between HIV prevention and care outcomes and three types of geospatial indicators: demographic characteristics (e.g., unemployment, poverty, racial composition), physical characteristics (e.g., public transportation, availability of HIV clinics), and social characteristics (e.g., crime, HIV prevalence, stigma). These authors found that physical characteristics have been most consistently linked with HIV outcomes [1]. For example, research in Chicago found neighborhoods with higher Walk Scores (i.e., neighborhoods that do not require a car to complete daily errands) were less likely to contain HIV-positive MSM, while neighborhoods with a larger proportion of vacant buildings were more likely to contain HIV-positive MSM (while controlling for individual-level risk factors); this indicates potential relationships between neighborhood disorder or instability, neighborhood isolation, and HIV risk [25]. Both of these studies highlight the importance of considering access to resources as a key aspect of neighborhood characteristics that may be related to HIV prevention and care outcomes.

Beyond neighborhood characteristics, it is also important to consider neighborhood-level patterns of affiliation. Black MSM showed lower levels of residential/socializing/sex neighborhood concordance than other racial/ethnic groups, which could indicate bridging between multiple high-risk environments [26]. High bridging can directly impact HIV risk, as it increases exposure to multiple risk environments and has been linked with specific sexual risk behaviors, such as group sex and sex in exchange for money [27]. This aligns with higher rates of sex in exchange for money among Black MSM [5]. Research with Chicago YMSM found that sexual ties between residents of different neighborhoods were significantly influenced by a dis-preference for Black neighborhoods and a preference for ties between neighborhoods with similar income (i.e., income homophily [28]). Thus, preliminary research in this area also highlights the importance of access to resources in shaping neighborhood-level patterns of affiliation. These dynamics of bridging multiple neighborhoods to get their needs met and navigating exposure to multiple high-risk environments may also lead to greater stress exposure for Black MSM.

## Current Study

**Fig. 1:**
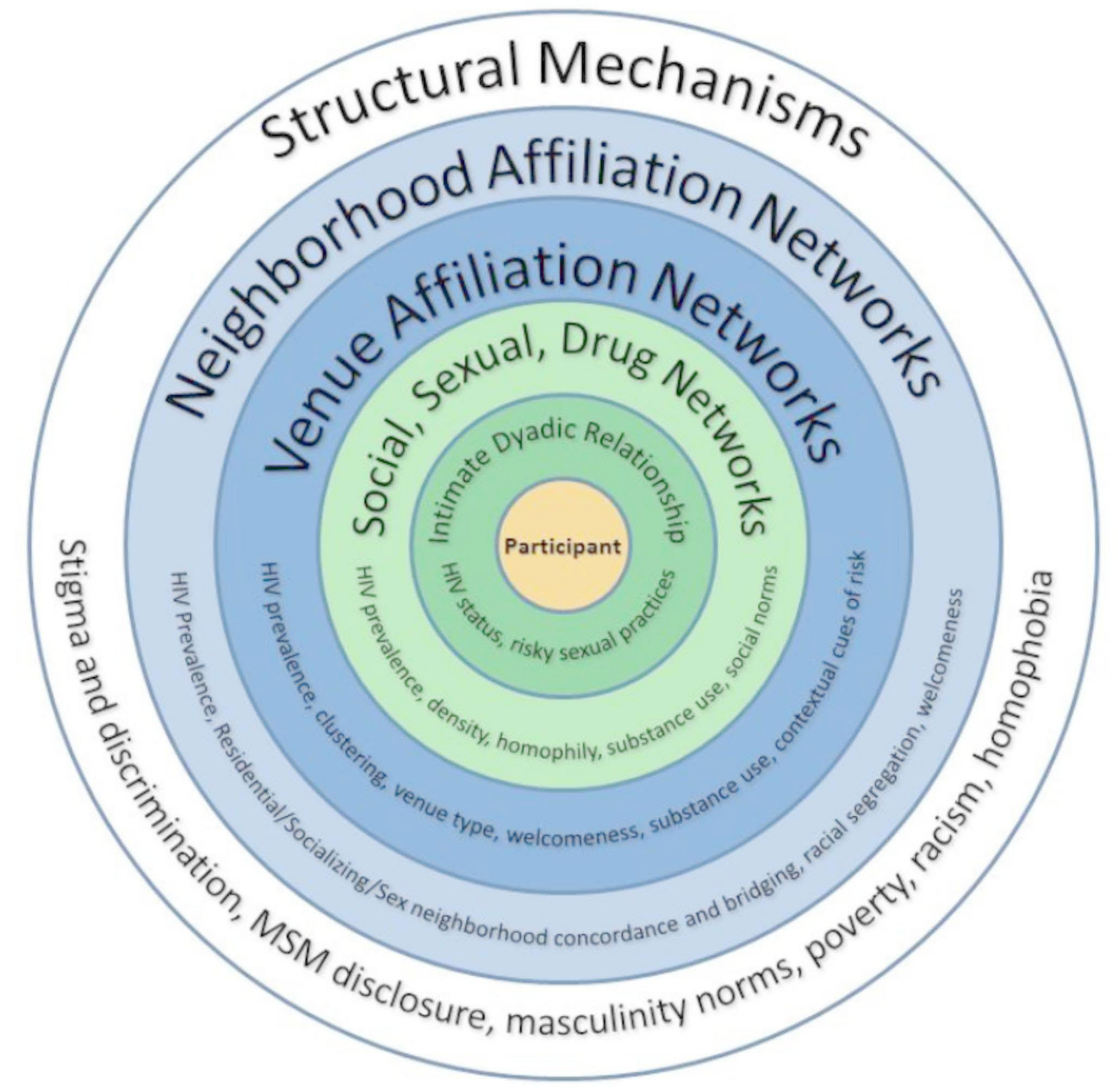
Potential network-level HIV risk factors based on the NIR model. The current study focuses on neighborhood-level affiliation networks

The Network-Individual-Resource Model and existing research on network- and neighborhood-level patterns that may drive racial disparities in HIV risk inspired the current study. For a conceptual overview of potential network-level HIV risk factors guided by NIR Model, please see Fig. 1. This study aimed to characterize the neighborhood-level networks of Black, White, and Latino YMSM in order to identify neighborhood affiliation dynamics that may drive racial disparities in HIV. Based on previous research [26], we hypothesized that Black YMSM would have higher levels of neighborhood bridging than White YMSM.

## Methods

Although network science provides a promising set of tools to characterize multilevel factors that may drive these disparities, quantitative methods alone are limited in their ability to capture the experiences behind YMSM’s neighborhood-level affiliations. Further, appropriately interpreting quantitative data describing these social and cultural contexts (i.e., the structure of individual, venue, and neighborhood affiliation networks) can be problematic without the incorporation of the voices, lived experiences, and insights of participants.

This mixed methods study utilized an explanatory sequential research design, in which quantitative data was visualized to identify patterns which were then explained using qualitative data collection and analysis [29]. Mixed methods projects are increasingly being used to address complex public health problems [30] and are especially suitable for projects in which a solely quantitative or qualitative approach is insufficient to develop a complete understanding of the research problem [31, 32]. Mixed methods approaches are especially promising for multilevel research as they allow both “zooming out” and “zooming in” to different levels of analysis [33]. Given the relative lack of research on multilevel risk factors driving racial disparities in HIV among YMSM, a mixed methods approach was especially suitable to the current study as it capitalizes on the potential of both qualitative and quantitative approaches to generate novel insights.

Mixed methods network research is especially promising to this end, as visualization of network data offers an intuitive way for participants to interface with data during qualitative interviews to elicit personal experiences and yield novel insights [34–37]. Network visualizations organize, structure, and display a great deal of complex information in a concise and coherent manner; this facilitates learning and understanding and can be especially useful for multilevel data [38–40]. This concrete representation of abstract concepts (e.g., relationships) anchors conversation and facilitates qualitative research [37, 41–43]. It also provides an excellent opportunity for researchers to involve community members, who have rich contextual and experiential knowledge that can greatly contribute to the formation of research questions and interpretation of findings [34].

In this study, quantitative network data from a parent study were used to generate visualizations of Black, White, and Latino YMSM’s neighborhood affiliations (Phase 1) which were then explained by participants in qualitative data collection and analysis (Phase 2). Participants were sampled from an ongoing longitudinal study of young men who have sex with men in Chicago [RADAR, U01DA0306939, PI: Mustanski; [44]. IRB approval was obtained from Northwestern University prior to conducting the study. Key points of data integration in the research design included nested sampling (in which a subset of participants in the quantitative parent study were recruited to complete Phase 2 qualitative interviews based on theoretical sampling criteria), connecting Phase 1 quantitative findings to Phase 2 qualitative data collection through the use of visualizations, and narrative integration of key patterns identified across Phases 1 and 2 during data analysis and reporting findings.

### Phase 1 Participants and Procedure

**Fig. 2:**
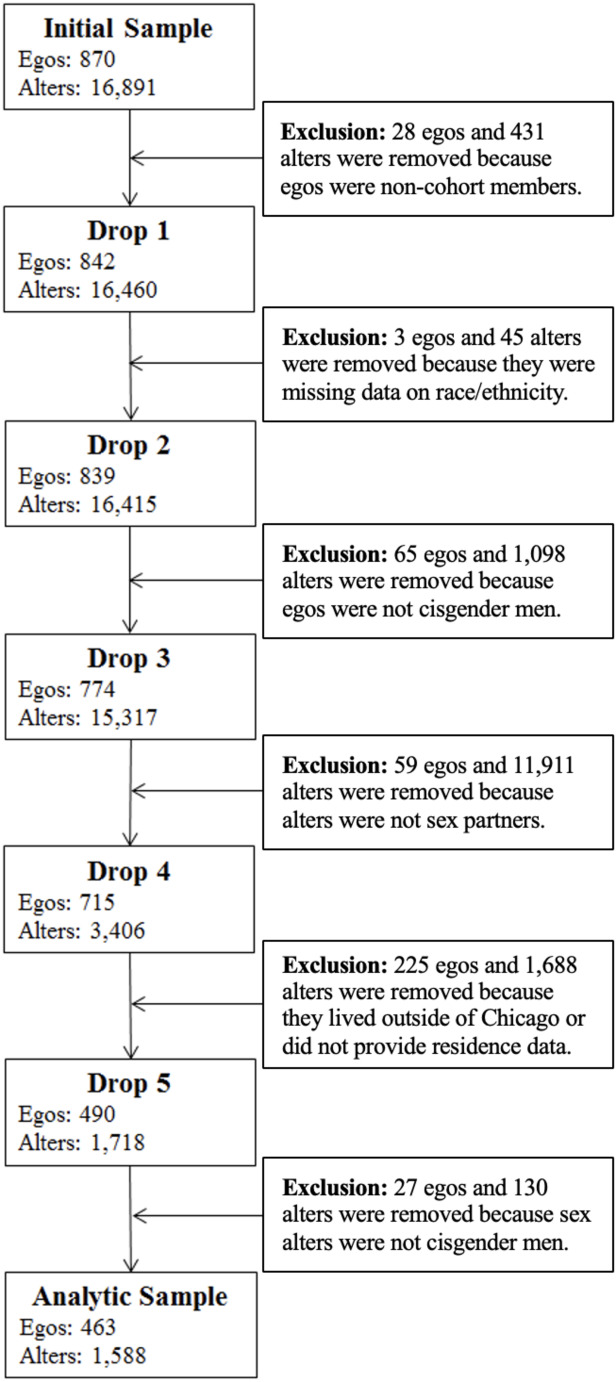
Exclusion criteria and data cleaning procedures for phase 1 analytic sample

In Phase 1, aggregated, deidentified network data collected from an initial sample of parent study participants (*n* = 463) and their sex alters (*n* = 1,588) were used by the first author to generate network visualizations at the venue and neighborhood levels. These 463 participants included initial seeds and as well as serious sexual partners and peers recruited into the longitudinal cohort. The sample was restricted to cisgender men who lived in Chicago and who reported at least one cisgender male sex partner who lived in Chicago. Sex partners included partners met through any method, including through apps and in-person. For an overview of the exclusion criteria and data cleaning procedures used to arrive at this analytic sample, see Fig. 2. For an overview of the ego demographics and sex partnerships of the Phase 1 analytic sample, see Table 1.

**Table 1:** Phase 1 analytic sample demographics and sex partnerships

Demographics	Egos (*n* = 463)	Sex alters (*n* = 1,588)
Ego age	21.5 (*SD* = 2.99, *Range* 16.01–29.74)	
*Ego race/ethnicity*		
Black	170 (36.7%)	475 (29.9%)
White	101 (21.8%)	449 (28.3%)
Latino	142 (30.7%)	493 (30.0%)
Other	50 (10.8%)	171 (10.8%)
*Ego sexual orientation*		
Gay	346 (74.73%)	
Bisexual	95 (20.52%)	
Queer	10 (2.16%)	
Unsure/Questioning	5 (1.08%)	
Other	7 (1.51%)	
*Homophilous sex partnerships*		
Black*Black		351 (22.10%)
White*White		302 (19.02%)
Latino*Latino		213 (12.41%)
Other*Other		20 (1.26%)
Total homophily		886 (55.79%)
*Non-homophilous sex partnerships*		
Black*White		61 (3.84%)
Black*Latino		132 (8.31%)
Black*Other		89 (5.60%)
Latino*White		254 (15.99%)
Latino*Other		68 (4.28%)
White*Other		98 (6.17%)
Total non-homophily		702 (44.21%)

**Fig. 3:**
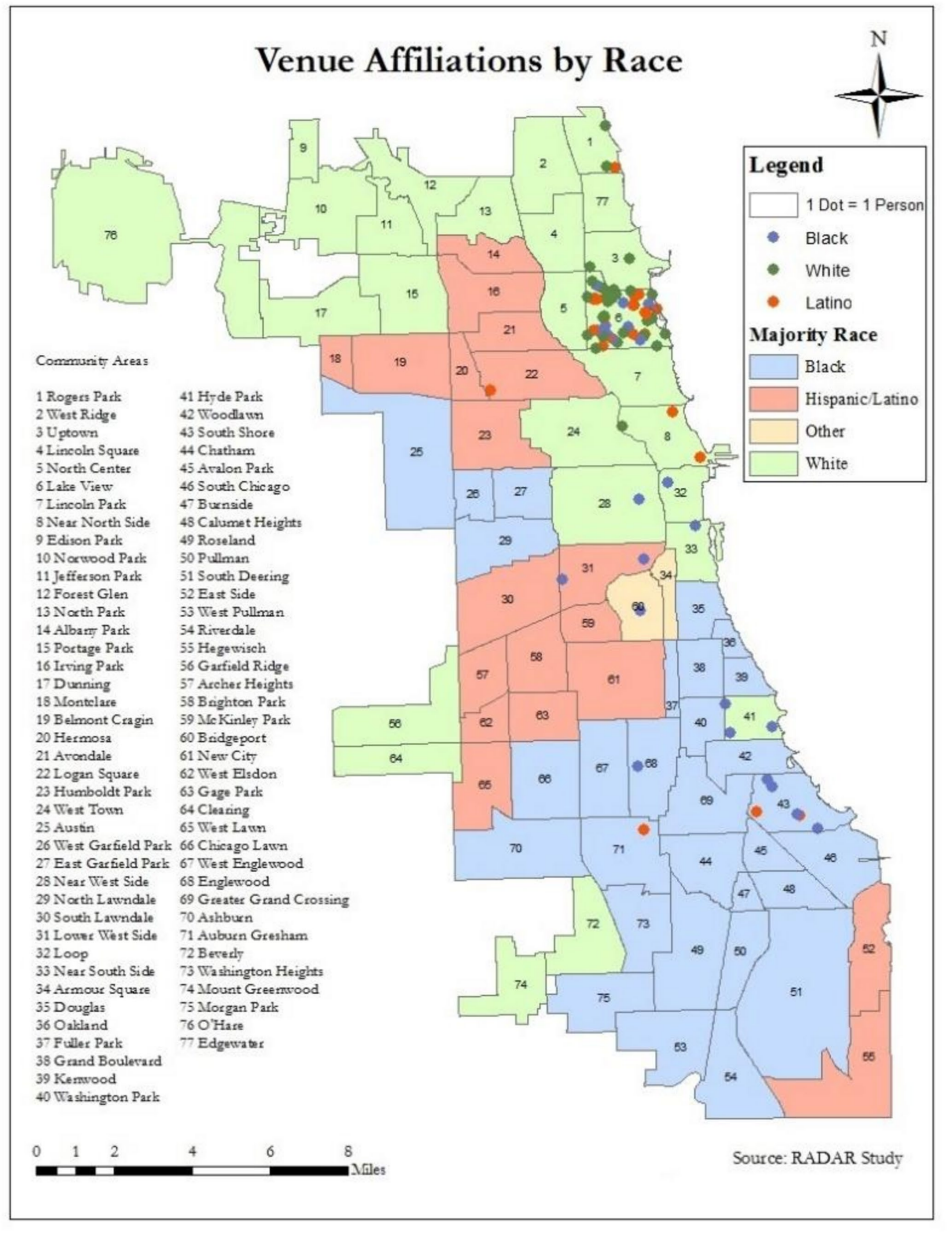
Locations of bars/clubs where RADAR participants met sex partners. Each dot represents one person who met a sex partner at a venue in that community area. Image depicts the community area where bars/clubs were located, not their exact location

**Fig. 4:**
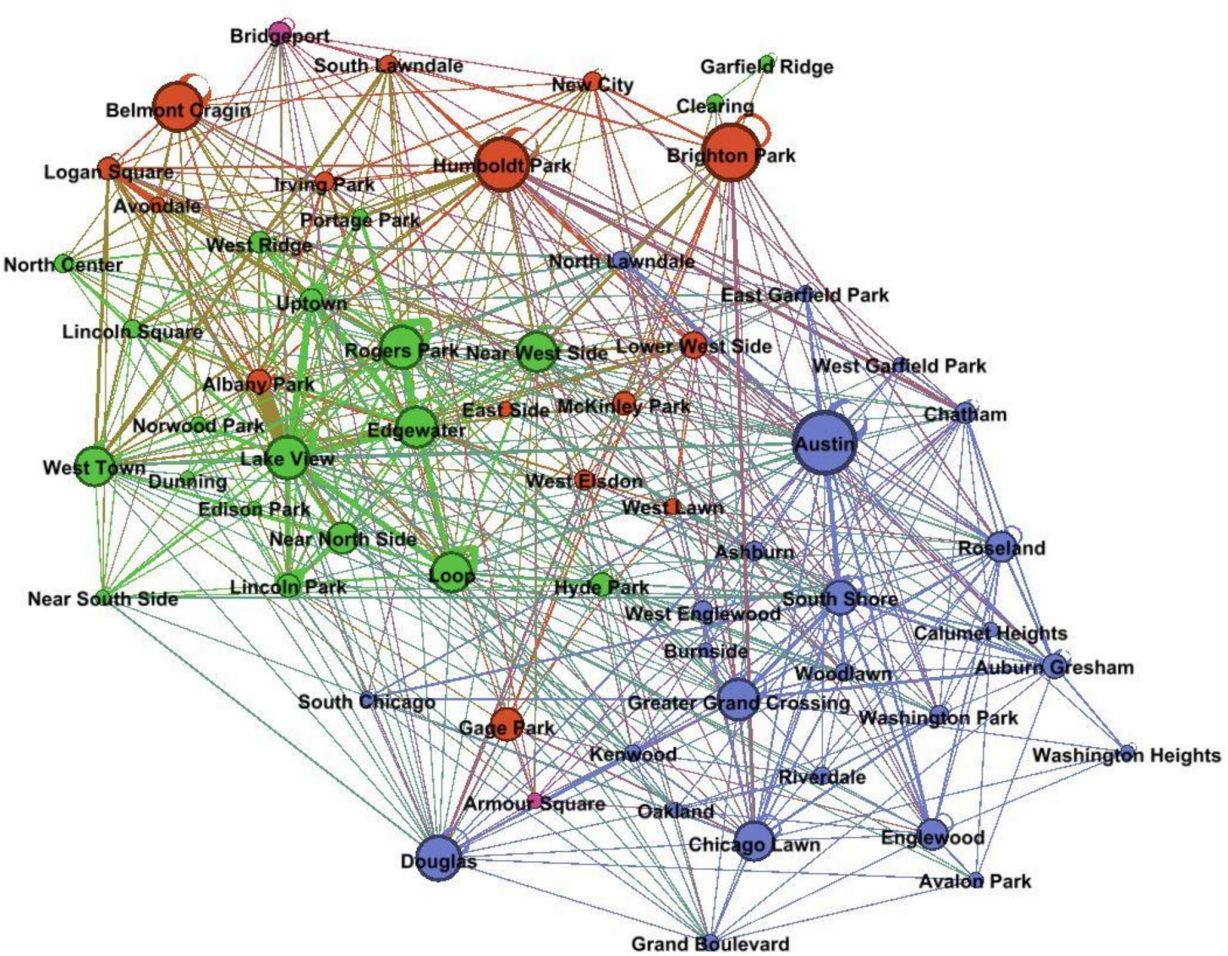
Neighborhood*neighborhood MSM sex connections. All node degrees  ≥ 5. Layout placed nodes that shared connections closer together, such that closeness between neighborhood nodes represents their overall of sexual connectedness, independent of geography. Node size represents betweenness centrality. Edge thickness represents edge degree. Color represents majority race using 2010 Census data (blue = Black, red = Latino, green = White) (Color figure online)

### Phase 1 Quantitative Data Analysis

Phase 1 aggregated and visualized individual, venue, and neighborhood network data in order to illustrate racial patterns in risk environments. Secondary network data from RADAR participants (*n* = 463) about their residential neighborhood, the residential neighborhood of their sex partners, and the location of the bars/clubs where they met sex partners was utilized in conjunction with 2010 Census data to construct aggregated illustrations of racial patterns at the venue and neighborhood levels. Incorporating Census data allowed us to display general neighborhood patterns non-specific to YMSM (e.g., residential racial segregation). Individual social, sexual, and drug networks were also visualized for substudy participants who scheduled a Phase 2 interview; however, these are not the primary focus of this study. Network visualizations were generated using Gephi [45] and geospatial visualizations were generated using ArcGIS Desktop [46]. Data visualization guidelines, including presenting information as simply and intuitively as possible, were followed [34, 38]. Overall, this phase focused on creating visualizations that depict racial differences in potential network-level HIV risk factors, such as differences in the types of places where YMSM met partners and geospatial patterns of affiliation and sexual partnership across Chicago. Visualizations were used as tools for reflection and discussion in Phase 2, similar to other mixed-methods approaches that utilize data visualization to guide the collection of qualitative information through interviews [35]. This study focused on two Phase 1 visualizations illustrating participants’ neighborhood-level affiliations.

### Phase 2 Participants and Procedure

In Phase 2, a subsample of 33 YMSM from the Phase 1 analytic sample was recruited to complete qualitative interviews using the visualizations created in Phase 1. Interviews were balanced to facilitate comparison across Black, Latino, and White participants. Both Black and Latino MSM show higher HIV prevalence compared to MSM of other races/ethnicities [2]. Additionally, as race-based stigma and discrimination and economic inequality may play a role in shaping risk environments, it is important to compare the experiences of racial/ethnic minority MSM with those of White MSM. Due to lower HIV prevalence rates and limited sample sizes for Asians, American Indians and Alaska Natives, and Native Hawaiians and other Pacific Islanders [2], participants in these racial groups were not included in the current study. Overall inclusion criteria for Phase 2 included the following: over 18 years of age, a member of one of the three targeted racial/ethnic groups, inclusion in the Phase 1 analytic sample, met theoretical sampling criteria (discussed below), and expressed willingness to complete an audio recorded interview. For an overview of the demographics of the Phase 2 qualitative sample, see Table 2.

**Table 2:** Phase 2 participants

Pseudonym	Age	Race/Ethnicity	Sexual orientation	HIV status
Hunter	28	White	Gay	Negative
Jared	26	Black	Gay	Positive
Lucas	24	Latino	Gay	Negative
Cesar	26	Latino/Black	Gay	Negative
Max	22	Latino	Gay	Negative
Luis	20	Latino	Gay	Negative
Javier	22	Latino	Gay	Negative
Gabriel	19	Latino	Gay	Negative
Jacob	20	White	Gay	Negative
Will	20	White	Gay	Negative
Cody	22	White	Gay	Negative
Anthony	20	Black	Gay	Positive
Steve	22	Black	Bisexual	Negative
Dave	21	White	Gay	Negative
Rodrigo	26	Latino	Gay	Negative
Brett	21	White	Gay	Negative
Colin	20	White	Unsure/Questioning	Negative
Hector	26	Latino	Gay	Positive
Randall	27	Black	Gay	Negative
Tyrell	26	Black	Bisexual	Positive
D’andre	24	Black	Bisexual	Negative
Louis	27	Black	Gay	Positive
Terell	28	Black	Gay	Positive
Chris	26	Black	Gay	Negative
Miguel	25	Latino	Gay	Negative
Jerome	20	Black	Gay	Positive
Joey	24	Latino	Gay	Negative
Brad	25	White	Gay	Negative
Luke	20	White	Queer	Negative
Michael	22	Black	Bisexual	Negative
James	21	White	Gay	Negative
Dustin	24	White	Gay	Negative
Danny	18	Latino	Gay	Negative

Interviews were conducted using a grounded theory approach. Data analysis was conducted in an iterative process alongside data collection, and theoretical sampling was used to aid the process of theory generation [47]. Initially, affiliation with neighborhoods identified in Phase 1 as potentially playing a key role in dynamics of HIV risk was one of the inclusion criteria for participation in the substudy. For example, neighborhoods with high betweenness centrality (i.e., that played a key role in bridging sex connections between different Chicago neighborhoods) were identified as recruitment targets. Additional participants were recruited using iterative theoretical sampling procedures based on criteria from Phases 1 and 2. The final subsample consisted of 11 White, 11 Black, and 11 Latino participants. Most (*n* = 27) identified as gay, four as bisexual, one as queer, and one as unsure/questioning. Participants’ average age was 23.1 (*SD* = 2.9; Range 18–28).

Interviews were structured according to the NIR Model [8]. Participants were first asked about their personal network, venue, and neighborhood affiliations, substance use behavior, and experiences of their intersectional identities. Next, they were shown the aggregated Phase 1 visualizations illustrating racial/ethnic patterns in RADAR participants’ network, venue, and neighborhood affiliations. The interviewer provided a brief explanation of each visualization, such as the meaning of visual characteristics like color. The interviewer also provided a brief overview of how to interpret network visualizations, including defining nodes and edges and explaining the meaning of network layout, clustering, and metrics like betweenness centrality. The interviewer checked with participants for understanding and addressed any questions before proceeding. Participants were asked about what patterns in the visualizations stood out to them as well as their reactions to and interpretations of these patterns (e.g., “How does that relate to your experiences?” and “Why do you think that is?”). Finally, participants were shown their individual network visualizations and asked about the types of relationships in their network as well as their responses to patterns illustrated in the visualizations.

The interview protocol underwent member checking [48] completed through feedback from the parent study Community Advisory Board and pilot testing with three participants. Interviews averaged 59 min (*Range*: 37–82 min) and were conducted as one-time, in-person visits by the first author between May and December 2017. Participants were compensated with $30 cash. Interviews were audio recorded, then transcribed and deidentified. Given that the first author’s identities as a White queer nonbinary person diverged from many study participants’ identities, a brief feedback form was used to anonymously assess participants’ experiences of the interview. Participants reported a high level of accuracy in their responses (*M* = 4.93) and comfort during the interview (*M* = 4.77).

### Researcher Positionality and Reflexivity

The first author identifies as a White queer nonbinary person and the second author identifies as a White half Puerto Rican genderqueer woman. Both authors have lived in and are most familiar with either majority-White neighborhoods on Chicago’s North Side or majority-Latine communities west of Chicago. Authors have both personal and professional experiences with Chicago’s LGBTQ + spaces and communities, but are more limited in direct personal experience with sexual minority men’s spaces and communities. Further, Whiteness fundamentally shapes our experiences within and outside of LGBTQ + community spaces, insulating us from many of the multilevel stressors that shape the experiences of sexual minority people of color. Alongside these personal layers, we have professional experience collaborating with Black and Latine sexual minority men’s communities (e.g., through community advisory boards, organizational affiliations, and other professional collaborations).

As clinical-community and counseling psychologists, we strove to take an ecological approach that incorporated a focus on multilevel social context and was grounded in values of diversity and social justice as well as feminist and constructivist frameworks. We addressed our positionality throughout the study in several ways. Prior to data collection, we met to identify sensitizing concepts and key ideas related to the topic of study [49]. These guided interview protocol development; however, we also included open-ended, general questions and modified the protocol as analysis developed to incorporate ideas beyond these sensitizing concepts. We worked to maintain reflexivity throughout, such as by discussing biases, journaling, and documenting team discussions [49]. This included discussing our interpretations of Phase 1 quantitative findings, which we allowed to inform Phase 2 qualitative analysis while also working to stay grounded in the qualitative data. For example, we noticed many of the same neighborhood-level patterns in the visualizations that participants identified. Although our qualitative coding of these dynamics was based on participants’ interview data, discussing how our interpretations of Phase 1 visualizations aligned with Phase 2 qualitative analysis helped us integrate quantitative and qualitative findings.

### Phase 2 Qualitative Data Analysis

The systematic approach to grounded theory outlined by Corbin and Strauss [47] was utilized to iteratively collect and analyze interview data. The first author worked with a single trained RA to complete coding. Transcripts were coded in several phases using Dedoose (Version 7.0.23; [50]). Interviews were first coded using open coding to summarize segments of text into concepts, which were then grouped using axial coding into higher-order categories representing the important ideas that emerged from the data. The research team reviewed and refined categories using constant comparison techniques to identify several categories to focus on as the core phenomena relevant to racial disparities in HIV. Categories continued to be refined using open coding and a codebook was created including descriptions and examples. All responses were then coded separately by both members of the research team with good inter-coder agreement (Cohen’s Kappa = 0.80) and any disagreements resolved through consensus. Consistent with the paradigm model [47], selective coding was used to identify conditions, actions/interactions, and consequences associated with categories. This process continued until theoretical saturation was reached after 33 interviews: no new relevant data emerged, categories were well developed and showed variation, and relationships among categories were established. This study focused on a single category related to neighborhood-level dynamics in participants’ affiliations across the city of Chicago. Following qualitative analysis, quantitative Phase 1 findings and qualitative Phase 2 findings were narratively integrated in the process of writing up study findings for dissemination.

## Results

### Phase 1: Visualizing Racial/Ethnic Patterns in Neighborhood Affiliation

Two visualizations were generated to illustrate both geospatial and social patterns of connectivity at the neighborhood level: a map that organized neighborhoods according to geographic proximity (Fig. 3) and a network image that organized Chicago neighborhoods according to their overall level of sexual connectedness (Fig. 4). Data from the 2010 Census was integrated into both visualizations. Figure 3 depicts the locations of the bars/clubs where Phase 1 participants met their sex partners using dots and shows the majority race/ethnicity of all 77 Chicago community areas using color. (Of note, only 5.8% of sex partners in the sample were met at venues; most [58.9%] were met through apps.) Dots were distributed at random within the boundaries of the community area where the bar/club was located and dot color indicates the participant’s race/ethnicity. Sex connections were concentrated in Lakeview, reflecting connections made at bars/clubs in North Halsted, a popular Chicago gay neighborhood. This community area also showed the highest racial/ethnic diversity in participants who met sex partners there. Although all bar/club connections for White participants were in majority White neighborhoods (*n* = 30), Black and Latino participants (*n* = 23 and 23) met their partners in all three types (i.e., majority White, majority Black, and majority Latino) of neighborhoods, and Black participants’ sex connections were more concentrated on the South Side. Overall, this image highlighted a pattern of White spatial insularity (i.e., White participants meeting partners at venues in majority-White neighborhoods) and Black and Latino spatial bridging (i.e., Black and Latino participants meeting sex partners around the city). Participants of all races/ethnicities met partners at venues in North Halsted, in a majority-white neighborhood on the north side of the city; this represented most of the venue-based sex connections in the sample.

Figure 4 depicts an aggregated network of sex connections between Phase 1 participants in different Chicago neighborhoods. The primary purpose of this visualization was to illustrate sexual connectedness between neighborhoods, irrespective of geographic distance. Given that Fig. 3 illustrated neighborhood-level racial segregation organized geospatially, Fig. 4 provided an important complement by illustrating neighborhood-level racial dynamics organized based on sexual connectedness. Each neighborhood was represented by a node, with node color depicting the majority race/ethnicity of the neighborhood. Node size represented betweenness centrality, a measure of network centrality based on path length that reflects the degree to which a given node connects other nodes in the network [51]. Nodes with higher betweenness centrality can be conceptualized as “hubs” connecting other neighborhoods and nodes with lower betweenness centrality can be conceptualized as more isolated. Edges between nodes indicated at least five sex connections in the Phase 1 sample, with thicker edges indicating more sex connections. Nodes were organized using several layout algorithms, including the Frutcherman Reingold algorithm, such that nodes grouped closer to each other shared a greater number of connections than nodes distributed further apart. Although these patterns of sexual connectivity may be informed by geographic distance (e.g., macro-level North-South White-Black segregation across the city means that it takes people a long time to travel between these neighborhoods), they also illustrate stronger racial segregation that we would expect based on geography alone (e.g., West Side majority-Black neighborhoods are clustered with South Side majority-Black neighborhoods rather than with more proximal majority-White or majority-Latino neighborhoods).

Neighborhood distribution in Fig. 4 illustrates racially homophilous clustering for majority Black and majority White neighborhoods, with more even distribution for majority Latino neighborhoods; this represents stronger Black-White neighborhood-level segregation with respect to the formation of sex partnerships. The neighborhoods with the highest betweenness centrality were primarily majority Latino neighborhoods (i.e., Belmont Cragin, Humboldt Park, and Brighton Park) with the addition of one majority Black neighborhood (i.e., Austin, a West Side neighborhood that borders a majority-White suburb and several majority-Latino neighborhoods). Thus, in addition to being more highly connected to both majority-Black and majority-White neighborhoods, majority-Latino neighborhoods also played a strong role in connecting neighborhoods across the network. Although a number of majority White neighborhoods showed moderate betweenness centrality (i.e., Lakeview, Edgewater, Rogers Park, West Town, the Loop, and Near West Side), they were well-connected to each other in an insular pattern (as reflected by the thicker edges connecting these nodes); this likely lowered their betweenness centrality as they were less likely to connect neighborhoods across the network. Overall, participants in majority Latino neighborhoods (and in Austin) were more likely to form sex connections that bridged across the network, while participants in majority White neighborhoods were sexually connected to other White neighborhoods. Phase 2 participants were shown an interactive version of this visualization in Gephi that allowed them to hover over any neighborhood in order to focus on its specific connections.

### Phase 2: Describing Racial Differences in YMSM’s Neighborhood Affiliations

The current study focused on a category of five concepts reflecting racial/ethnic differences in YMSM’s neighborhood affiliation dynamics related to HIV risk (see Table 3). The following language is used to describe the number of participants who contributed to concepts: *all* denotes 33 interviewees, *most* denotes 26–32, *many* indicates 16–25, *some* indicates 6–15, and *few* signifies five or fewer. For concepts described with respect to specific racial/ethnic groups, the following language is used: *all* denotes 11 interviewees, *most* denotes 8–10, *many* indicates 5–7, *some* indicates 3–4, and *few* signifies 1–2. Responses reflect both participants’ reactions to and interpretations of the neighborhood level visuals as well as their direct personal experiences.

**Table 3:** Frequency of categories and concepts by race/ethnicity

Category	Concept	Black	Latino	White	Total
Racial/ethnic	Differences in YMSM’s neighborhood affiliation dynamics	11	11	11	33
	Racial segregation perpetuates fundamental inequities	11	11	11	33
	White YMSM cluster in North Side spatial insularity	4	9	10	23
	Black and Latino YMSM bridge multiple neighborhoods	11	8	9	28
	YMSM of different races mix in North Halsted	9	6	10	25
	Latino neighborhoods are gentrifying and becoming more White	2	6	4	12

Counts reflect participants’ discussion of both their own and others’ experiences, including interpretation of the visualizations or observation of others; thus, they should not be interpreted to reflect participants’ direct personal experiences with a particular concept

**Racial/ethnic segregation perpetuates fundamental inequalities.** All participants (*n* = 33) identified that *racial segregation perpetuates fundamental inequities*, noting that racial segregation was characterized by both demographic separation and resource inequality. Participants described this as a defining characteristic of the landscape of the city and identified ways in which this impacted their affiliations with and experiences of Chicago neighborhoods. Cesar, a Black/Latino participant who lived in Humboldt Park, said:

I feel a lot of things about the racial segregation. Well not the segregation but the opportunities in certain neighborhoods and the education system in certain neighborhoods and the overall lack of certain neighborhoods. In some neighborhoods the upkeep is better than other neighborhoods. And other neighborhoods are just left for shot…You can see that when you cross certain borders.

Resource inequalities related to racial segregation were identified with respect to streets and sanitation, education, law enforcement, economic opportunity, community violence and physical safety, the concentration of commercial and recreational facilities, and access to LGBTQ specific resources. Participants’ experiences of racial segregation were also related to all other neighborhood affiliation dynamics. Given these pervasive associations and the fact that all participants identified it as an important neighborhood dynamic, racial segregation (and associated resource inequality) is best understood as foundational to YMSM’s neighborhood-level affiliations around Chicago.

**White YMSM cluster in North side spatial insularity.** Many participants (*n* = 23) reported spatial insularity, in which people largely spent time within the same few neighborhoods that were geographically close to each other. This dynamic was primarily characterized by *White YMSM clustering in North Side spatial insularity*. For example, Dustin, a White participant who lived in Rogers Park, said, “It’s helpful for me to see the data in this way actually. Because it just continues to reinforce for me how much more of the city there is to experience and what a small pocket I’m living in that feels like my complete world of Chicago, which is not the same shared experience with the majority of other neighborhoods in the city.” Jacob, a White participant who lived in Lincoln Park, described a similar experience:

I mean I pretty much only ever stay here [pointing to the North Side]. I live here, I have school here, I work here. Sometimes I have school here [pointing to the Loop]. It’s just kind of like, I live and work in the same little north-south track right there… partially because of routine but also because of convenience I just live and work off the Brown Line… I never really have a need to go outside of my little green bubble.

Many White participants reported being completely unfamiliar with neighborhoods on the West and South Sides of the city, with the exception of gentrified neighborhoods like Wicker Park and Logan Square. For example, James, a White participant who lived in Lakeview, said:

I’ve never heard of most of these areas that are here in the blue. Like Austin? I’ve never, it’s like the biggest one and I’ve never heard of it. Is that in Chicago?… What I’m familiar with is these areas right here, and so I’m White, so it makes sense that this is what I’m familiar with. And I’m not familiar with any of these areas at all. Which makes sense because I wouldn’t associate myself with these areas.

Although most White participants reported insularity within the concentration of majority-White neighborhoods on the North Side, Will reported insularity within majority-White neighborhoods even though he lived in Hyde Park on the majority-Black South Side, where he attended school. He said, “I mostly stick around campus… the people from campus go on trips a lot up north to the neighborhoods… Probably the big ones are like Logan Square, Lincoln Park, and Wicker Park I guess. Just cause those are like the cool gentrified neighborhoods… So I don’t necessarily spend a lot of time in places that are like really heavily skewed not White.” Will’s experience illustrates how a neighborhood’s racial composition (and associated economic and resource concentration) may be more important than geographic proximity in shaping YMSM’s neighborhood-level affiliations. Overall, White participants described neighborhood insularity in majority-White neighborhoods.

Although this neighborhood dynamic was most prominent for White participants, it was also reported by some Black and Latino participants. For example, Steve, a Black participant who lived in Roseland, described how he tended to stay on the South Side because he “never really wanted to travel out west because… that’s not my area, so I don’t really know anything about it. Even though I sometimes travel out west, I still tend to get lost… I know the South Side. It’s programmed in my brain so I know where I’m going.” Steve’s experience was in some ways unique because he was heavily involved with an LGBTQ+-affirming fraternity on the South Side, which was where he spent virtually all of his free time; without this affirming space close to home, it is possible that Steve would have felt a greater need to access LGBTQ resources and community, as discussed below.

**Black and Latino YMSM bridge multiple neighborhoods.** In contrast, most participants (*n* = 28) reported *Black and Latino YMSM bridge multiple neighborhoods*. Black and Latino participants expressed greater familiarity with a wide variety of neighborhoods in different parts of the city. Michael, a Black participant who lived in Austin, said he spent time “a little bit of all over Chicago. Everywhere. From the south suburbs to the west suburbs, be up north, took a trip to Carbondale a couple times… Just be wherever and all over.” Rodrigo, a Latino participant who lived in South Shore, described a similar experience: “I’m actually spread out throughout Chicago. I have friends all over Chicago. Some of the places include Hyde Park, Beverly, which is far south. I spend time in Progress [Bar], usually, but that’s Boystown, but it’s usually just Progress [Bar]. And the North Side. So it kind of varies.” These experiences bridging between multiple neighborhoods with different racial/ethnic compositions on different sides of the city stand in stark contrast to White participants’ experiences of neighborhood insularity.

Neighborhood bridging was often linked to the concentration of LGBTQ + resources in North Halsted (formerly known as Boystown), experiences of homophobia in the neighborhoods where participants lived, or other structural factors such as community violence and neighborhood resources. Tyrell, a Black participant who lived in South Shore, described how LGBTQ + community and resources shaped his neighborhood affiliations:

When I went to high school, I ran into a couple of people who were gay. Them being older than me, they adopted me as their younger brother. And from there, one of them introduced me to the North Side and to Boystown for the centers. And from the centers I just met so many people and like, I just been moving around… Like I’ve stayed in La Casa Norte… Another resource that I got from being inside of the medical Part D health program at Howard Brown… is that they sent me to a place on the West Side that helped me get housing and helped me get a job and training skills.

Anthony, a Black participant who lived in Englewood, echoed the importance of North Halsted in accessing LGBTQ + community. He described how he actually met some of his friends who lived on the far South Side in North Halsted: “Like I’ve never heard of north, Boystown or nothing, until one of my friends they told me that’s where a lot of LGBT community is. So I went up there, I met a lot of my friends up there.” The concentration of LGBTQ + resources and community in North Halsted was a powerful anchor that helped shape Anthony’s connections to other Black gay men on the South Side, even though these relationships were formed on the North Side. Overall, these complex affiliations with many different neighborhoods around the city of Chicago, including to strategically access LGBTQ + resources, characterized Black and Latino participants’ experiences of neighborhood bridging.

**YMSM of different races/ethnicities mix in North Halsted.** Many participants also described *YMSM of different races mixing in North Halsted* as an important neighborhood dynamic (*n* = 25). Participants identified North Halsted as a hub of bars/clubs, LGBTQ + resources, and LGBTQ + community that drew YMSM from around the city. Hector, a Latino participant who lived in Belmont Cragin, said simply: “You do see more intermingling cause it’s Boystown.” D’andre, a Black participant who lived in Brighton Park, said, “A lot of people met in Boystown… That’s kind of the spot.” Brett, a White participant who lived in Lakeview, echoed this: “It’s not surprising to me that Lakeview seems pretty mixed… everyone’s there, everyone’s at the party.” As these participants describe, the concentration of LGBTQ + resources and community in North Halsted drew participants from a variety of neighborhoods. Thus, although North Halsted is located in Lakeview, a majority-White neighborhood, participants reported experiencing a high degree of racial/ethnic mixing there.

**Latino neighborhoods are gentrifying and becoming more White.** Finally, some participants (including over half of Latino participants) described how *Latino neighborhoods are gentrifying and becoming more White* (*n* = 12). Several Latino participants who grew up on Chicago’s West Side described watching the neighborhoods where they lived gentrify over time. Hector, a Latino participant who grew up in Humboldt Park, described his experience:

Literally my neighborhood was always Hispanic, and like three Black houses on my block… And now, we have on the same block, we have three people that just moved in, and they just built a house. So they demolished the old houses we had, right next door to me too. And it’s like, it’s weird cause you start seeing that… You’re like, “Oh, now that there’s White people here, you see more running, and that’s what I wanted to do when I was younger.” It’s like, I just want to run through Humboldt Park and not get shot at, or get looked at wrong cause you’re like, “Why the hell are you running?” (Laughs).

Other Latino participants who grew up in Humboldt Park and Pilsen (in the Lower West Side)described similar changes, including new construction, an influx of White residents, and Latino residents being pushed out of the neighborhood and into the suburbs. Miguel, a Latino participant who lived in Lakeview, commented that gentrification was consistent with the bridging role Latino neighborhoods played in the neighborhood-level sexual network image. He went on to say, “White people move to Hispanic neighborhoods and interact with Hispanic neighborhoods because they’re seen as, you know, not entirely safe but not as dangerous as Black neighborhoods.” Several participants made similar observations in response to the neighborhood map, noting that Latino neighborhoods tended to be geographically located between White and Black neighborhoods.

A few Black participants described similar patterns of gentrification in Austin, a majority-Black neighborhood on Chicago’s West Side. Austin is bordered by majority-White suburb Oak Park to the west and majority-Latino Humboldt Park (another gentrifying neighborhood) to the east. In this way, it is geographically more similar to majority-Latino neighborhoods that tend to border White and Latino neighborhoods than to majority-Black neighborhoods, which tend to be more dramatically segregated. Overall, participants highlighted how gentrification is particularly impacting Latino neighborhoods, making them more White over time.

## Discussion

The purpose of this two-phase, mixed methods study was to examine racial/ethnic differences in the neighborhood-level affiliations of YMSM that may shape HIV risk. Both quantitative and qualitative findings illustrated neighborhood-level patterns of White spatial insularity, strong Black-White segregation, and Black and Latino spatial bridging. These bridging neighborhood dynamics are consistent with previous research findings, which found lower residential/socializing/sex neighborhood concordance [26] and high clustering around a few venues [19–23] among Black MSM. Neighborhood-affiliation dynamics for Latino participants appeared to fall “in-between” patterns for White and Black participants: geographically, majority-Latino neighborhoods are often located between majority-White and majority-Black neighborhoods; with respect to neighborhood-level sex connections, majority-Latino neighborhoods played an important role in connecting majority-White and majority-Black neighborhoods, which were otherwise highly segregated.

These patterns of White spatial insularity, strong Black-White segregation, and Black and Latino spatial bridging have powerful potential to create and maintain racial disparities in HIV. White spatial insularity reflects White MSM’s tendency to meet sex partners in majority-White, lower HIV-prevalence venues and neighborhoods with fewer contextual risk factors (e.g., lower walkability and income, higher vacant buildings and proportion of Black residents [1, 25]) and greater resources (e.g., LGBTQ + spaces, HIV prevention). Strong Black-White neighborhood segregation effectively separates Black and White MSM in different parts of the city, which likely helps to maintain population differences in HIV and other health burdens. Further, even when MSM of different races come together (e.g., in LGBTQ + spaces in North Halsted), racial stigma may function to socially and sexually isolate Black MSM, contributing to higher rates of racial homophily in this group [52, 53]. Thus, although Black and Latino YMSM in this study bridged multiple environments, they did so in the context of both racial segregation and racial stigma, which appear to function in ways that isolate and concentrate HIV burden among Black MSM. Importantly, recent work [10] has demonstrated that the experiences of Black-Latino MSM tend to mirror the experiences of Black MSM and experiences of White-Latino MSM tend to mirror the experiences of White MSM. Had the current study used this combination of racial and ethnic identities, it is possible the bridging patterns observed in Latino YMSM would have been specific to Black-Latino YMSM, with White-Latino YMSM more closely mirroring White YMSM.

The NIR model underscores the importance of considering the interplay between individuals, mental and tangible resources, and their network-level contexts in order to understand HIV risk. These complex, multilevel dynamics may help explain why Black YMSM have higher rates of HIV despite lower individual risk behaviors. In this study, racial segregation fundamentally shaped YMSM’s neighborhood-level affiliations, highlighting these links between individuals (e.g., participants’ race/ethnicity), resources (e.g., both general and LGBTQ + resources), and network-level contexts (e.g., patterns of White insularity and Black and Latino bridging). Study participants expressed strong recognition of the extent to which racial segregation is not only characterized by demographic separation, but is also associated with inequities in community resources and health outcomes. Participants named specific resource inequities related to community violence and physical safety, city services such as streets and sanitation, public transit options, experiences with law enforcement, community resources such as businesses and parks, and LGBTQ+-specific resources such as bars and social service agencies. These disparities were especially pronounced for Black participants who lived on the South Side, but were also present for Black and Latino participants who lived on the West Side.

A substantial body of research has documented the powerful influence of racial segregation on health disparities, particularly for Black communities [54, 55]. Racial segregation is associated with poorer housing quality and access to medical care, increased exposure to community violence and homicide, and reduced educational and employment opportunities, which negatively impact SES. For those in under-resourced neighborhoods, segregation may increase health risk behaviors (including drinking and smoking) as means of escape or seeking relief from chronic stressors while inhibiting health promoting behaviors (including physical exercise and eating fresh foods) given lack of access to community resources [55]. Although these differences in individual risk behaviors are profound, study findings highlight the importance of examining how racial segregation shapes network-level contexts, particularly for health disparities that are not explained by differences in individual risk behaviors like HIV. In the current study, key neighborhood affiliation dynamics of profound Black-White segregation, Black and Latino spatial bridging, and White spatial insularity may function to disproportionately concentrate HIV burden among Black YMSM.

White participants showed patterns of spatial insularity, where they primarily lived, worked, studied, and socialized in majority-White neighborhoods on the North Side. Participants identified feeling comfortable, accepted, and safe with respect to their intersectional identities in these neighborhoods [56] and reported easy access to community and LGBTQ + resources, including bars and clubs in North Halsted. White participants were most familiar with majority-White North Side neighborhoods, often expressing that they had not ever visited or even heard of many neighborhoods in other parts of the city, particularly majority-Black neighborhoods on the South Side. Many White participants described never feeling a need to leave majority-White neighborhoods as all of their needs were met in these spaces, reflecting greater experiences of accessibility and ease. These insular neighborhood-level affiliation patterns likely also shape White YMSM’s sex connections, as reflected in the densely sexually connected cluster of majority-White neighborhoods in Fig. 4. Given lower prevalence of HIV among White YMSM compared to other racial/ethnic groups, this pattern of neighborhood-level insularity likely functions to keep White YMSM in spaces where they are relatively less likely to form a sexual partnership with an HIV-positive partner.

Racial segregation was most profound in separating majority-White and majority-Black neighborhoods, with majority-Latino neighborhoods often falling in-between. Black and White neighborhoods were the most separated geographically, with majority-White neighborhoods concentrated on Chicago’s North Side and majority-Black neighborhoods concentrated on Chicago’s South Side. They were also the most separated with respect to neighborhood-level sex connections, as illustrated by the strong clustering of White and Black neighborhoods on opposite sides of the sex network in Fig. 4. Beyond demographic separation, the resource inequalities between White and Black neighborhoods were the most pronounced with respect to both community and LGBTQ + resources. By contrast, majority-Latino neighborhoods were spatially located between White and Black neighborhoods and Latino participants reported a pattern of gentrification over their lifetimes, which resulted in Latino neighborhoods becoming more White. Further, majority-Latino neighborhoods were sexually connected to both majority-White and majority-Black neighborhoods, as reflected in their placement throughout the network in Fig. 4. This pattern of profound Black-White segregation (both geospatial (Fig. 3) and sexual (Fig. 4)) likely functions to keep Black YMSM in spaces where they are relatively more likely to form a sexual partnership with an HIV-positive partner. However, in contrast to the insularity demonstrated by White participants, Black participants’ neighborhood affiliations were characterized by both segregation and bridging, which were driven at least in part by inequities in community and LGBTQ + resource allocation.

Both Black and Latino participants demonstrated high spatial bridging: they were more likely to spend time in different neighborhoods around the city, which varied in their racial/ethnic composition, the extent to which participants felt safe and accepted with respect to their intersectional identities [56], and their access to community and LGBTQ + resources. Many Black and Latino participants identified spending time in North Halsted because they felt more comfortable and affirmed with respect to their sexuality there than they did in their home neighborhoods [56]. As Fig. 3 and qualitative findings illustrate, North Halsted played an important role in bringing together YMSM of different racial/ethnic groups thorough LGBTQ+-specific venues, and many Black and Latino participants reported traveling from around the city to access these affirmative spaces. Thus, in contrast to White participants (who reported having many kinds of needs easily met within the same cluster of majority-White neighborhoods on the North Side), Black and Latino participants traveled to neighborhoods around Chicago in order to access resources and navigate identity-based stigma.

Given the difference in ease of access to various Chicago places and neighborhoods for Black and Latino YMSM who lived on the West Side as compared to Black YMSM who lived on the South Side, racial segregation likely influences the social and spatial environments and HIV risk of these populations in different ways. While Black and Latino participants who live on the West Side may negotiate more micro-level segregation given their relative ease of access to different neighborhoods, Black participants who live on the South Side may negotiate more macro-level segregation given the concentration of Black neighborhoods on the South Side and the relative lack of transportation to the rest of the city. In turn, this may shape the opportunity structure within which YMSM form social and sexual relationships, with neighborhoods on the South Side more likely to be isolated and clustered together and neighborhoods on the West Side associated with higher bridging. These patterns were reflected in Fig. 4, which showed high betweenness centrality of West Side majority-Latino neighborhoods and Austin (a majority-Black West Side neighborhood) with relatively lower betweenness centrality among majority-Black South Side neighborhoods.

Consistent with these findings, research has found that macro-level patterns of segregation contribute more to Black-White segregation than Hispanic-White segregation, and also that macro-level segregation contributes more to segregation in local environments in the Midwest than in other regions of the country [57]. Research also has documented that a dis-preference for Black neighborhoods contributes to sexual tie formation among YMSM in Chicago over and above the effect of income homophily [28] and that neighborhoods with less access to transportation and higher levels of neighborhood disorder were more likely to contain HIV-positive MSM over and above individual level risk factors [1, 25]. Given racialized differences in exposure to risk and protective factors at the community level related to these patterns of segregation [54, 55, 57–59], these macro-level segregation dynamics are more likely to isolate Black YMSM– particularly on Chicago’s South Side– in environments that place them at greater risk for health disparities more generally and for HIV specifically.

### Implications for Research

Research is needed that examines the direct impact of racial segregation and associated resource inequalities on health as well as examining how it creates opportunity structures that further shape and segregate HIV risk environments. Although segregation is less extreme for Latino populations, there is less research on how racial segregation impacts this population [54, 55]. In the current study, the neighborhood-level affiliations of Latino participants were often somewhat “in-between” those of Black and White participants. Given myriad pathways through which racial segregation negatively impacts health, research is needed that develops conceptual frameworks linking structural neighborhood segregation to specific health outcomes like HIV [54]. Multilevel, network, and mixed methods hold strong promise in this regard. For example, the current study illustrated patterns of Black and Latino bridging, strong Black-White segregation, White insularity, and racial mixing in North Halsted that provide greater insight into how racial segregation may be experienced differently for Black, White, and Latino YMSM in ways that impact their HIV risk. Overall, findings highlight the importance of research on foundational, structural forces like racial segregation that underlie racial disparities in HIV.

### Implications for Public Policy

Findings underscore the importance of HIV prevention and treatment policies that attend to the different ecological contexts experienced by YMSM of different races/ethnicities. Black and Latino participants in this study reported traveling to a range of neighborhoods to meet their needs, including accessing LGBTQ + and community resources. Research has documented that a lack of HIV testing and prevention services in the neighborhoods where Black MSM live likely contributes to racial disparities in HIV [1, 60]. This aligns with findings in the current study, which highlight how strong Black-White segregation characterized participants’ neighborhood experiences. Further, given that participants in the current study were interviewed on the North Side, it is possible that study findings do not represent the experiences of Black YMSM on Chicago’s South Side who are less likely to bridge multiple neighborhoods and who may particularly benefit from HIV prevention resources localized in their residential neighborhoods. For example, access to PrEP can both prevent HIV- YMSM from becoming HIV + when exposed to the virus and prevent HIV + YMSM from transmitting it if their viral loads are undetectable. Policies should incentivize the creation of these resources in underserved communities, in addition to working to dismantle racial segregation and resource inequality more broadly.

### Strengths and Limitations

The current study has several noteworthy strengths. Its mixed-methods approach and explanatory sequential design integrated quantitative survey, network, and geospatial data with qualitative interview data to provide a rich and nuanced perspective on the lived experiences of White, Black, and Latino YMSM in Chicago. Further, the process of visualizing parent study data and presenting these findings back to a subset of YMSM in the parent study enabled the direct participation of study participants in the interpretation of quantitative study findings. This allowed for the incorporation of participants’ expertise, which helped to deepen and contextualize patterns identified in the quantitative data. Overall, participants reported positive experiences of the interview, related both to their experience with the interviewer and the opportunity to view and respond to the data visualizations. These experiences of trust and comfort likely contributed to the validity of study data.

This study followed recommendations for conducting intersectional psychological research. First, it focused on particular intersections of identity (i.e., race/ethnicity, gender, and sexual orientation), thus contributing to incremental advances in intersectionality research [61]. Second, it moved beyond demographics to explore the relationships between intersectional identities and specific health-related constructs, including HIV risk [62]. Third, it acknowledged the context-dependent nature of identities given the interplay between people and environments [63, 64]. Fourth, it focused on how people’s experiences of marginalization may differ rather than on summing the effects of multiple, separate marginalized identities [65]. Fifth, questions were phrased in a way that allowed for the interdependence of identities rather than asking participants to consider their identities separately or rank them in salience or importance [62, 63].

However, this study also had several important limitations. First, this study focused on the particular context of YMSM’s experiences in Chicago neighborhoods. Although sex partners met on apps were included in this study, we did not directly discuss participants’ experiences on apps; this examination was included in a separate study. The patterns identified here may differ for YMSM in other urban centers in other areas of the country, in rural areas, or international contexts. Chicago is characterized by strong North-South White-Black segregation with Latino neighborhoods buffering between them, but other cities have different patterns of racial segregation, including more micro-level segregation (e.g., New York, Houston), strong East-West White-Black segregation without buffering Latino neighborhoods (e.g., Atlanta), and greater representation of majority-Latino neighborhoods (e.g., Miami, Houston). Research on school segregation has found that White students are overrepresented in suburban and rural areas and under-represented in urban areas, while the opposite is true for Black and Latino students. Further, patterns of racial segregation and associated resource inequality persist in suburban and rural areas, even if the specific dynamics of the segregation may differ from urban environments [66]. Future research should examine multilevel factors that may shape racial disparities in HIV in these contexts. Second, although the parent study sought representation of YMSM from around the city of Chicago, there was potential sampling and retention bias given that the parent study site was located at Center on Halsted, an LGBTQ community center on the North Side of the city. Further, although the neighborhood-based sampling strategy utilized in the current study helped to ensure representation from different community areas, it included more participants from the West and North Sides than the South Side. These characteristics of the sample could have resulted in under-representation of Black YMSM who live in majority-Black neighborhoods on Chicago’s South Side and who are less likely or able to travel to LGBTQ spaces on the North Side. Third, quantitative data visualizations were largely generated using participants’ responses to questions about where they met their sex partners. Given the nature of this question, quantitative findings do not necessarily represent where participants go with the *intent* to meet sex partners, and also say little about where they go to spend time more generally. Fourth, interviews were time-limited and designed to last no longer than one hour, which left a limited amount of time for exploring or discussing each visualization or topic. Fifth, although some participants were living with HIV, the interview did not explicitly ask about their experiences with HIV, including HIV stigma. Participants were also not asked about engagement with HIV testing and prevention services, including use of PrEP.

## Conclusion

Overall, study findings illustrated important differences in the neighborhood-level affiliations of White, Black, and Latino MSM, which may in turn drive racial disparities in HIV. Deeply entrenched patterns of racial segregation and resource inequality shaped participants’ exposure to community-level risk and protective factors as well as their affiliations and movement around the city. Black and Latino participants reported bridging different neighborhoods to avoid community stressors and access resources (including LGBTQ + resources), while White participants reported patterns of insularity in majority-White neighborhoods. These experiences illustrate the power of how participants’ intersectional identities relate to broader societal structures in shaping their experiences at multiple levels, such that YMSM of different races/ethnicities essentially experience different “Chicagos.”

Findings from the current study reinforce the importance of multilevel influences on racial disparities in HIV among YMSM. Although research has documented that racial/ethnic differences in individual risk behaviors are not responsible for the disproportionate impact of HIV on Black YMSM [5], national approaches to HIV prevention and treatment continue to target individuals, such as increasing HIV testing and treatment as well as prevention behaviors like use of condoms and pre- and post-exposure prophylaxis [6]. Study findings suggest that deeper, structural changes are needed to address resource inequalities associated with racial segregation and the pervasive impacts of stigma and discrimination around YMSM’s intersectional identities [56].
